# Methods for Analyzing Alternative Splicing and Its Regulation in Plants: From Gene‐Specific Approaches to Transcriptome‐Wide Studies

**DOI:** 10.1111/ppl.70639

**Published:** 2025-11-18

**Authors:** Stavros Vraggalas, Oussama Guennich, Boushra Shalha, Christos Bazakos, Hélène S. Robert, Olha Lakhneko, Sotirios Fragkostefanakis

**Affiliations:** ^1^ Molecular and Cell Biology of Plants, Institute of Molecular Biosciences Goethe University Frankfurt Frankfurt am Main Germany; ^2^ Mendel Center for Plant Genomics and Proteomics, CEITEC MU—Central European Institute of Technology Masaryk University Brno Czech Republic; ^3^ National Centre for Biomolecular Research, Faculty of Science Masaryk University Brno Czech Republic; ^4^ Institute of Plant Breeding and Genetic Resources, ELGO DIMITRA Thermi Greece; ^5^ Institute of Plant Genetics and Biotechnology, Plant Science and Biodiversity Centre, Slovak Academy of Sciences Nitra Slovakia

**Keywords:** alternative splicing, plant RNA biology, RNA‐binding proteins, splicing regulation, transcriptome‐wide analysis

## Abstract

Precursor messenger RNA (pre‐mRNA) splicing is a fundamental mechanism of gene regulation that influences both mRNA abundance and proteome diversity. In plants, alternative splicing plays a critical role in coordinating development and enabling responses to environmental stress. This process is tightly regulated by the spliceosome and associated splicing factors, which recognize conserved sequence motifs in pre‐mRNAs to guide intron removal and exon joining. In this review, we summarize and compare experimental approaches used to analyze both the regulation of alternative splicing and the splicing profiles of genes, spanning from gene‐specific assays to transcriptome‐wide methods. Gene‐specific techniques, such as minigene assays, transient expression systems, electrophoretic mobility shift assays, and isothermal titration calorimetry, provide insights into the molecular interactions between splicing factors and their RNA targets. To identify RNA‐binding partners of splicing factors, or splicing factors that interact with a specific RNA, we discuss high‐throughput methods that can be applied in vivo and in vitro. By comparing these approaches, we highlight their advantages, limitations, and applications in plant biology. Understanding alternative splicing regulation is essential for deciphering its role in plant adaptation to environmental challenges, with potential implications for crop improvement strategies.

## 1 Introduction

Precursor messenger RNA (pre‐mRNA) splicing is a conserved post‐transcriptional process that removes introns and joins exons to produce mature mRNAs. In eukaryotes, this reaction is catalyzed by the spliceosome, a complex composed of five small nuclear ribonucleoproteins (snRNPs) and associated proteins. The spliceosome recognizes four conserved intronic elements: the 5′ and 3′ splice sites, the branch point, and a polypyrimidine tract (Dubrovina et al. 2013; Kováčová et al. 2020; Cheng et al. 2023).

Alternative splicing (AS) allows a single gene to produce multiple transcript isoforms by selectively joining different exon–intron combinations. Common AS types include exon skipping, intron retention (IR), alternative 5′/3′ splice sites, and mutually exclusive exons (Filichkin et al. 2010; Reddy et al. 2013). In plants, IR is the most frequent and is linked to stress responses and development (Szakonyi and Duque 2018). AS diversifies the transcriptome and can regulate gene expression by influencing mRNA stability, localization, and translation (Reddy et al. 2013).

AS is prevalent in plants, with more than 60% of multiexon genes undergoing AS in *Arabidopsis thaliana* and even higher percentages in crops like rice, maize, and soybean (Song et al. 2020; Martín et al. 2021). It is essential for processes like embryogenesis, flowering, and circadian rhythms (Lee et al. 2013; Szakonyi and Duque 2018), and it modulates responses to abiotic and biotic stress through dynamic shifts in splicing patterns, which may trigger the synthesis of truncated proteins or elimination of the aberrant RNAs by nonsense‐mediated decay (NMD; Kalyna et al. 2012; Filichkin et al. 2018; Hu et al. 2020). AS regulation involves *cis*‐elements (intronic or exonic motifs) and *trans*‐acting factors such as Serine/Arginine‐rich splicing factors (SRSFs) and heterogeneous nuclear ribonucleoproteins (hnRNP). Splicing factors are influenced by developmental and environmental cues that, in turn, modulate splice site selection (Matlin et al. 2005; Wang et al. 2015).

To study AS, researchers use gene‐specific assays for high‐resolution analysis and transcriptome‐wide methods to capture global splicing patterns. Both have unique strengths and limitations and currently several approaches are available, varying in the level of expertise, equipment, and cost required. This review outlines experimental strategies for AS detection and regulation in plants. We compare these methods in terms of resolution, scalability, and relevance to plant development and stress responses to support future research on transcriptome plasticity. In several cases, we refer to in vivo methods that have been scarcely used or not yet established in plants, providing alternative paths for future methodological development and offering opportunities to expand our understanding of splicing regulation in plant systems. In the same direction, methods used for detecting protein–nucleic acid interactions that have not yet been widely applied in RNA splicing studies are also discussed, highlighting their potential to uncover novel regulatory mechanisms and broaden the experimental toolbox available for plant RNA biology.

## 2 PCR‐Based Methods to Analyze AS at a Gene‐Specific Level

AS events can be efficiently detected using reverse transcription PCR (RT‐PCR), a method that provides rapid and gene‐specific insights into splicing patterns. However, reliable detection and interpretation depend on careful experimental design and validation steps. PCR amplifies regions across exon–exon junctions or intron‐containing segments, and different splice isoforms generate amplicons of distinct sizes. PCR requires several optimizations to provide reliable results.

The amplicon size alone does not confirm the exact nature of the splice event. Amplification of the full length of the cDNA, typically followed by Sanger sequencing of the amplicons, is essential to identify splicing events and splice junctions (SJs) with high confidence, especially when databases with splice variants are not available. This approach might require high‐resolution gel electrophoresis to distinguish amplicons of similar size. The quantitative analysis of splice variants requires high‐fidelity polymerases to reduce the introduction of errors and ensure reproducibility. The RNA that is used for cDNA synthesis must be of high quality, as degraded RNA may result in incomplete or misleading splice variant detection and quantification. It is also essential to eliminate genomic DNA that could lead to intron‐containing false positives. Quantitative analysis is more reliable when performed for a specific event rather than on the full‐length cDNA, as the amplification efficiency might vary among products with considerably different lengths.

To ensure reliable semi‐quantitative analysis of splice variants by PCR, the number of amplification cycles must be optimized to remain within the exponential phase of the reaction. This can be achieved by performing a cycle titration experiment, running identical reactions with increasing cycle numbers, and selecting the lowest cycle at which all splice variants are detectable while avoiding saturation. This approach enables a semi‐quantitative comparison of isoform abundance, although it provides only approximate results and is less commonly used in current practices. More accurate quantification can be achieved using capillary gel electrophoresis such as with the Agilent Bioanalyzer which allows precise sizing and quantification of PCR products; this approach has been successfully applied in splice‐variant studies using DNA high‐sensitive microcapillary chips (Mauger et al. 2015). Band intensities from gels may still be analyzed with imaging software (e.g., ImageJ; Abràmoff et al. 2004), but data should be interpreted with caution. Maintaining equal cDNA input and consistent PCR conditions across samples is essential for reproducibility.

Complex splicing events, especially those which generate RNAs with only a few nucleotides' difference, cannot be analyzed by agarose or even acrylamide gel electrophoresis due to the low resolution of these methods. Capillary fragment analysis is a highly sensitive technique allowing quantification of alternative splice isoforms with high precision. In this approach, RT‐PCR products are fluorescently labeled (e.g., using a fluorescein‐tagged primer), separated on a capillary electrophoresis system (e.g., ABI PRISM 3130xl Genetic Analyzer), and analyzed using fragment analysis software (e.g., GeneMapper by Applied Biosystems). This method allows resolution and quantification of splice isoforms that differ by as little as a few base pairs, and it has been used to create a panel of alternatively spliced genes of interest for simultaneous high‐resolution analysis (Simpson et al. 2008).

Quantitative PCR (qPCR)‐based approaches allow relative quantification using ΔCt or ΔΔCt methods to compare isoform abundance between treatments or genotypes. ΔCt follows the relative changes between two isoforms across different samples, whereas the ΔΔCt method allows the comparison of isoform expression levels between treatments or genotypes, normalized to a reference gene (or sets of reference genes) and a control sample (Livak and Schmittgen 2001). When absolute quantification is required, it is possible to generate standard curves from cloned isoforms or use digital droplet PCR (ddPCR). In ddPCR, the sample is partitioned into thousands of nanoliter‐sized droplets, each serving as an independent PCR microreaction. Following thermal cycling, droplets are scored as positive or negative for amplification, and the absolute copy number of each splice isoform is calculated using Poisson statistics, without the need for standard curves or reference genes. ddPCR is highly sensitive and reproducible, and allows the quantitative measurement of AS events, even for low‐abundance isoforms or analysis of complex samples (Song et al. 2023).

## 3 Transcriptome‐Wide Profiling of AS Using Short‐ and Long‐Read Sequencing Platforms

The ability to profile AS at a transcriptome‐wide scale has been transformed by advances in high‐throughput sequencing technologies, with both short‐ and long‐read platforms offering complementary advantages (Table 1). Short‐read Illumina RNA‐seq has become the standard for AS analysis due to its high base‐calling accuracy and depth, enabling detection of SJs and isoform quantification through alignment to reference genomes. It has been particularly effective in model plants with well‐annotated genomes (Filichkin et al. 2010). However, the reconstruction of full‐length transcripts from short reads is computationally challenging and often inaccurate for complex splicing events or non‐model species lacking high‐quality reference genomes.

**TABLE 1:** Comparative overview of RNA sequencing platforms for alternative splicing analysis.

Feature	Short‐read (e.g., Illumina)	Long‐read	
		PacBio SMRT	Oxford Nanopore Technologies (ONT)
Template	cDNA	cDNA	Native RNA or cDNA
Library preparation	Reverse transcription & PCR	Reverse transcription & PCR	Optional reverse transcription; direct sequencing of RNA possible
Read length	Short (50–300 bp)	Long (1–10 kb+)	Long (1–100 kb; lower quality on very long RNAs)
Sequencing biases	High: Incomplete cDNA synthesis PCR bias (e.g., GC content)	Moderate: Incomplete cDNA synthesis PCR Improved full‐length capture using consensus reads	Low (for direct RNA): No amplification bias Avoids reverse transcription artifacts Still prone to truncated reads (especially 5′ ends)
Base accuracy	Very high (> 99.9%)	Very high (HiFi reads 99.95%)	Moderate (~96%; lower in direct RNA mode)
Quantitative power	High	Moderate	Moderate
Isoform resolution	Low to medium (reconstructed computationally)	High (full‐length cDNA isoforms)	High (direct isoform‐level resolution; captures epitranscriptomic marks)
Additional features	Good for quantification & expression profiling	Ideal for full‐length transcript discovery	Captures RNA modifications; avoids RT‐PCR bias
Main limitations	Can't resolve complex isoforms Misses many splice variants	Costly Requires high‐quality cDNA	Higher error rates Low throughput Truncated reads (5′ bias due to motor protein stalling)
Best suited for	Accurate quantification in model species	Isoform discovery in both model/non‐models	Detailed isoform structures, RNA modification studies in complex or under‐annotated genomes

These limitations are addressed by long‐read sequencing platforms such as PacBio SMRT (Pacific Biosciences Single Molecule Real‐Time) and Oxford Nanopore Technologies (ONT; Rhoads and Au 2015; Bayega et al. 2018; Zhao et al. 2019). These platforms capture full‐length isoforms, enhancing the resolution of complex AS patterns and facilitating novel transcript discovery and genome annotation. For example, PacBio sequencing uncovered up to 54,600 high‐quality isoforms per rice cultivar under abiotic stress, ~40% of which were novel (Schaarschmidt et al. 2020), and revealed over 7000 new AS events in sorghum (Clavijo et al. 2017), and more than 107,000 unique isoforms in sugarcane (Hoang et al. 2017). ONT studies identified over 60% novel isoforms in *Brassica napus* and 67% novel AS events in rice reproductive tissues (Li et al. 2021, 2022). ONT direct RNA‐seq also allows the detection of native RNA modifications (Garalde et al. 2018). Although it can be constrained by lower throughput and higher error rates (Moldován et al. 2018).

Despite their power, long‐read methods can miss low‐abundance transcripts due to reduced depth and higher costs. Thus, a combined strategy is increasingly recommended: Long reads provide isoform‐level resolution, while short reads offer accurate quantification (Yang et al. 2021). Emerging technologies such as Kinnex (PacBio) bridge the gap between long‐ and short‐read sequencing by enabling high‐throughput, cost‐effective, full‐length transcript sequencing (PacBio 2024). This development further enhances the potential of integrated approaches to decode complex AS patterns, especially in non‐model systems.

### 3.1 Sequencing Data Processing and Analysis

**FIGURE 1:**
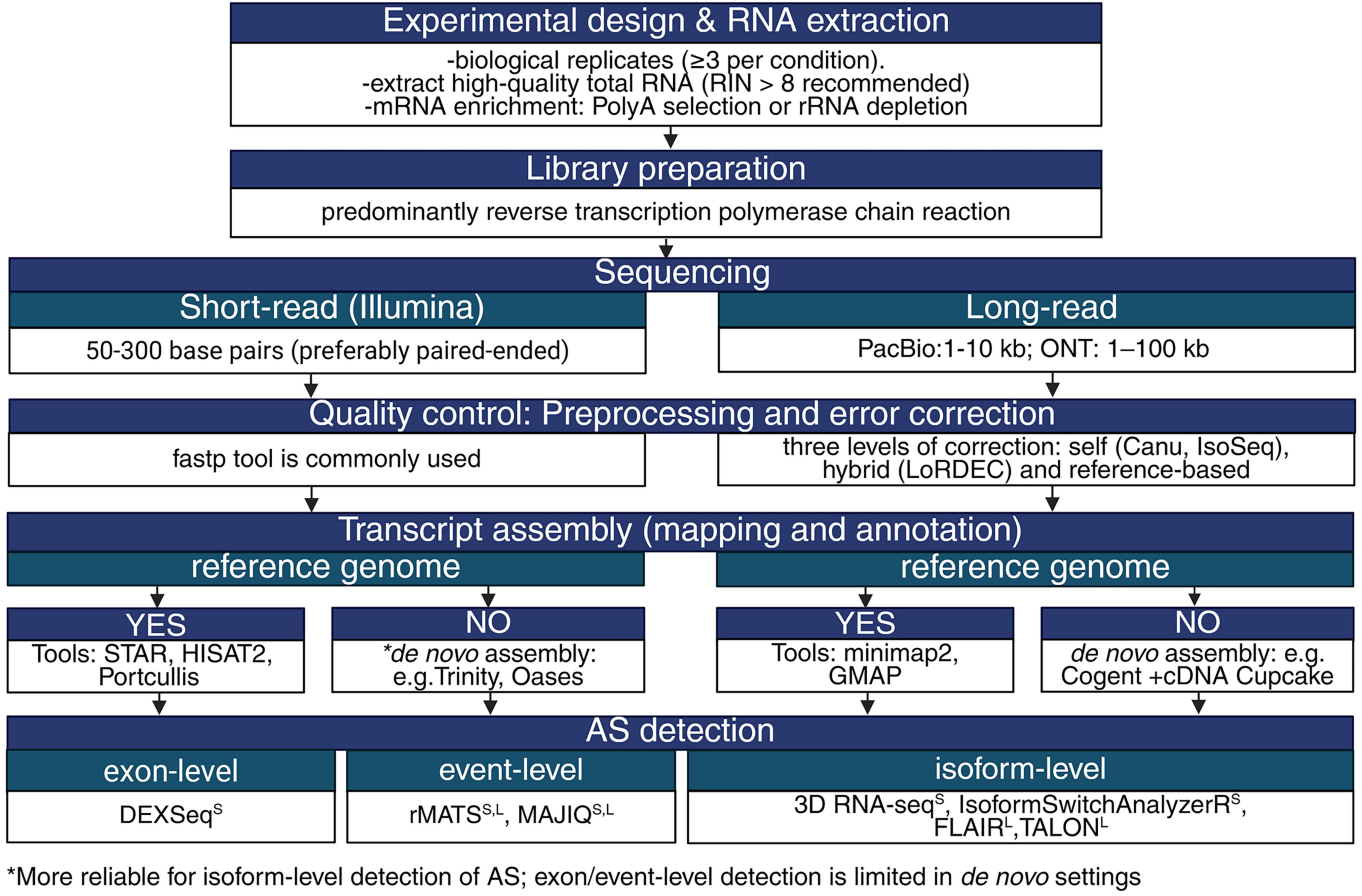
RNA‐seq data analysis pipelines with commonly used bioinformatic tools in alternative splicing (AS) analysis. Abbreviations: kb, kilobase (1000 base pairs); L, tool suitable for long‐read RNA‐seq; ONT, Oxford Nanopore Technologies; PacBio, Pacific Biosciences; RIN, RNA integrity number (created with BioRender); S, tool suitable for short‐read RNA‐seq.

A representative pipeline, illustrated in Figure 1, outlines the general steps of transcriptomic analysis, from sample preparation to short‐ and long‐read data processing, highlighting key parameters and commonly used tools. High‐quality RNA is critical for RNA‐seq, particularly for AS analysis. For AS detection, library prep often uses polyA selection for coding transcripts or rRNA depletion to retain non‐coding RNAs. mRNA is reverse‐transcribed into cDNA and PCR‐amplified, or directly sequenced in ONT workflows. A key consideration is the trade‐off between biological replicates and sequencing depth. Although more replicates improve statistical power, greater depth increases sensitivity for rare isoforms. A minimum of three biological replicates per condition is essential to perform reliable inferential analyses. Optimal sequencing depth for AS analysis remains poorly defined in the literature. Unlike gene‐level expression studies, AS detection requires higher read depth to cover SJs and isoform‐specific regions. Sequencing depth needs also vary by species, depending on genome size, transcriptome complexity, ploidy, and gene architecture (Conesa et al. 2016). Junction saturation analysis (e.g., via RSeQC) assesses whether read depth sufficiently captures splicing complexity (Wang et al. 2012). This involves rarefaction of aligned reads to determine how many SJs are detected at increasing depths. A plateau in junction detection indicates sufficient coverage, ensuring reliable AS profiling without unnecessary sequencing.

#### 3.1.1 Pipelines for Analysis of AS by Short‐Read RNA Sequencing

Short‐read sequencing remains the most common method for AS detection. Reads are pre‐processed with tools like fastp (Chen et al. 2018), then mapped using reference‐guided approaches (e.g., STAR, HISAT2) for annotated genomes (Dobin et al. 2013; Kim et al. 2019), or de novo assemblers (e.g., Trinity, Oases) for non‐model plants (Grabherr et al. 2011; Schulz et al. 2012). De novo assembly may introduce artifacts, but integrated tools like Mikado (Venturini et al. 2018) or EvidentialGene (Gilbert 2019) can improve transcript reconstruction. In polyploid species, combining assemblers followed by filtering, enhances isoform recovery (He et al. 2015).

#### 3.1.2 Pipelines for Analysis of AS by Long‐Read RNA Sequencing

Long‐read RNA sequencing platforms such as PacBio Iso‐Seq and ONT enable full‐length transcript profiling, which is crucial for resolving complex AS events (Table S1). Due to their relatively high error rates, these technologies require specific preprocessing and correction steps prior to AS analysis (Rhoads and Au 2015; Bayega et al. 2018; Moldován et al. 2018; Zhao et al. 2019). The pipeline begins with basecalling, which transforms raw signal data into nucleotide sequences using platform‐specific algorithms (Rhoads and Au 2015; Zhao et al. 2019). This is followed by adapter and barcode trimming to remove technical sequences to produce clean reads (Rhoads and Au 2015; Zhao et al. 2019). Quality filtering eliminates low‐quality or chimeric reads, which is essential to avoid misassembled transcripts (Moldován et al. 2018; Zhao et al. 2019).

Full‐length transcript detection follows, identifying reads that span complete transcripts from 5′ to 3′ ends, enabling isoform‐level resolution (Bayega et al. 2018). Subsequently, error correction is performed using one of three strategies: self‐correction (consensus from overlapping reads), hybrid correction (short‐read guidance), or genome‐based correction through spliced alignment (Bayega et al. 2018; Moldován et al. 2018). In the clustering and polishing step, reads are grouped into transcript isoforms, and consensus sequences are refined. Some workflows skip clustering to preserve fine‐scale splicing differences, such as micro‐exons and NAGNAG events (Bayega et al. 2018).

Corrected reads are then aligned to a reference genome using splice‐aware mappers optimized for long‐read characteristics (Wu and Watanabe 2005; Dobin et al. 2013; Li 2018). Aligned reads (BAM format) are grouped by shared SJs to form transcript isoforms, with those differing at junctions classified as AS events (Wang et al. 2018; Zhang et al. 2019). If no reference genome is available, a genome‐free approach can reconstruct coding regions directly from long reads (Tseng et al. 2016). This method has been validated in non‐model species like *Gossypium austral* and *Astragalus membranaceus* , where isoforms identified by this approach showed strong concordance with short‐read RNA‐seq data (Hoang et al. 2017; Li et al. 2017).

A final enhancement involves SJ‐centric correction, where long‐read‐derived SJs are filtered based on support from short‐read data, increasing specificity without compromising isoform diversity. This method has been successfully implemented in *A. thaliana* (Li et al. 2017). The final outputs of the pipeline typically include aligned read files (BAM) and transcript annotations (GTF/GFF), which are used for downstream AS quantification and visualization.

### 3.2 Detection of Alternative Splicing Events

Multiple bioinformatics tools detect AS by comparing aligned RNA‐seq data between conditions (e.g., stress vs. control). AS can be analyzed at the exon, event, or isoform levels, each offering a different resolution—from specific exon changes to full transcript isoform shifts.

Exon‐level detection focuses on the inclusion or exclusion of individual exons within transcripts. It is quantified using read counts overlapping the exon body or junctions that support its inclusion or skipping. The comparison between conditions identifies exons with significant changes in usage, regardless of the broader splicing context. A commonly used approach for exon‐level analysis divides genes into exon bins, quantifies reads per bin, and tests for significant differences in exon usage across conditions using statistical models (Anders et al. 2012; Leshkowitz et al. 2022). It is particularly effective for detecting subtle changes in exon inclusion.

Event‐level detection approaches, such as rMATS (Shen et al. 2014) and MAJIQ (Vaquero‐Garcia et al. 2016), detect and quantify known and novel AS events using the Percent Spliced‐In (PSI/*Ψ*) index. They differ in PSI calculation, reflecting the complexity of AS types each reports. rMATS is a widely used tool for detecting AS events between two conditions with replicates, accounting for biological variability from alignment files and an annotation (Shen et al. 2014). It extracts junction and exon body reads, uses genome annotations to identify known and novel AS events, and quantifies the five basic types (exon skipping, alternative 5′ splice sites, alternative 3′ splice sites, mutually exclusive exons, and IR) using:

$$\Psi =\frac{\text{Inclusion Reads}}{\text{Inclusion Reads}+\text{Skipping Reads}}$$

Changes between two samples are reported as ΔPSI.

MAJIQ identifies AS by building de novo splice graphs from short‐ or long‐read alignments, independent of existing annotations (Vaquero‐Garcia et al. 2016). It detects local splicing variations (LSVs), splicing events defined at a single splice site connected to multiple alternative junctions. For each junction, usage is quantified as a Percent Spliced‐In (*Ψ*) value, enabling the detection of complex, non‐canonical splicing patterns.

$$\begin{array}{c} \Psi ⱼ=\frac{\text{reads}\;{\text{supporting junction}}_{j}\;}{\sum_{\left\{k=1\right\}}^{\left\{N\right\}}\text{reads}\;{\text{supporting junction}}_{k}\;} \end{array}$$

This approach allows detection of complex, multi‐junction splicing events that fall outside the five canonical AS types.

Isoform‐level detection assesses changes in the relative usage of full‐length transcript isoforms, each defined by unique exon and SJ combinations. By comparing isoform ratios across conditions based on transcript‐level expression, this approach identifies subtle or complex splicing changes that may be missed at the exon or event level. Importantly, it detects splicing shifts even when total gene expression remains unchanged.

For each isoform, the Percent Spliced‐In (PSI) value is calculated based on transcript and gene abundance in every condition:

$$\Psi =\frac{\text{Expression of isoform}}{\sum \text{Expression of}\;\mathrm{all}\;\text{isoforms from the same gene}}$$

3D RNA‐seq (Guo et al. 2021) and IsoformSwitchAnalyzeR (Vitting‐Seerup and Sandelin 2019) are widely used tools for short‐read RNA‐seq data, while FLAIR (Tang et al. 2020) and TALON (Wyman et al. 2020) are the most commonly applied tools for long‐read RNA‐seq, following similar analytical principles.

## 4 Decoding Protein–RNA Interactions in Alternative Splicing

**FIGURE 2:**
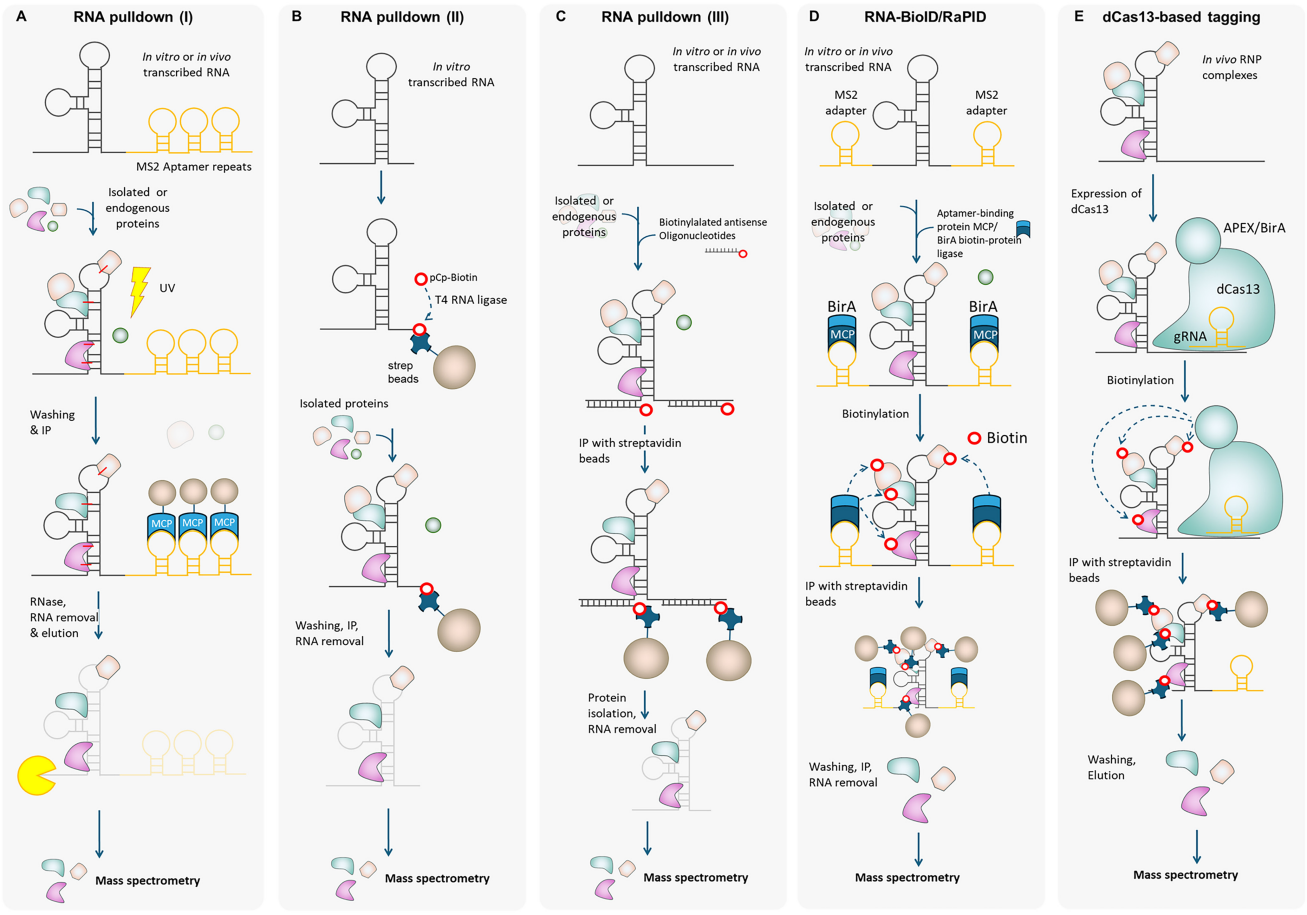
Overview of main RNA‐centric methods to identify RBPs bound to specific RNAs. From left to right: (A) RNA pulldown (I) techniques using RNA bait, tagged with RNA aptamers such as MS2 (in AP‐MS (RNA Affinity Purification‐MS) with two independent tags). Pulldown is done with MS2 coat protein (MCP) beads. (B) RNA pulldown (II): In vitro transcribed RNA, labeled with biotin, is incubated with native protein extracts. RNA‐protein complexes are captured on streptavidin‐coated beads, washed, and eluted. This approach captures proteins with direct or high‐affinity interactions, though it may include non‐specific binders and lacks in vivo context. (C) RNA pulldown (III): In vivo pulldown assays such as ChIRP‐MS/TRAP‐MS are hybridization‐based techniques using biotinylated antisense oligonucleotides to target endogenous RNAs in crosslinked cells. The RNA and associated proteins are enriched using streptavidin beads. ChIRP‐MS typically uses dense tiling probes, whereas RAP‐MS employs long probes and higher stringency, often yielding lower background. Both methods preserve native structures and modifications but may co‐purify indirect interactors depending on the crosslinking strategy. (D) RNA‐BioID/RaPID are proximity labeling techniques employing tagged RNAs (e.g., MS2‐tagged) co‐expressed with binding proteins fused to promiscuous biotin ligases (e.g., APEX2, BirA*, or BASU). Nearby proteins are biotinylated in vivo, allowing their enrichment. These methods allow detection of both stable and transient interactors in living cells but require engineered RNA tags. (E) Cas13‐based tagging (e.g., CARPID, RiboPro, CRIUS): CRISPR‐based methods use catalytically inactive Cas13 (dCas13) fused to a biotin ligase. Guided by a gRNA, dCas13 binds the endogenous RNA of interest and biotinylates proximal proteins in vivo. In all cases (A–E), enriched protein is eluted, purified and analyzed by MS. Western blot analysis can be used for all techniques if antibodies are available.

**FIGURE 3:**
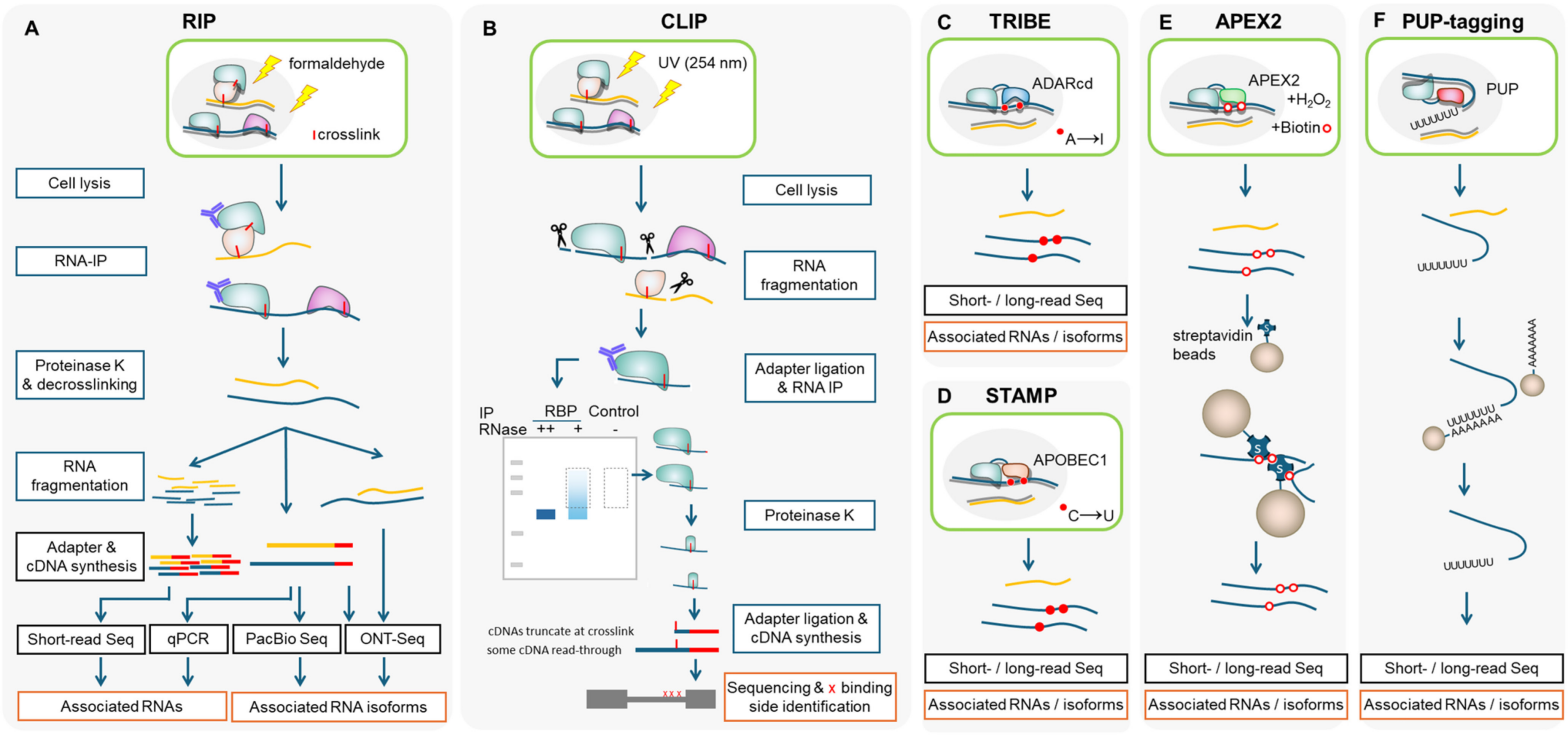
Protein‐based approaches for identifying RNA‐binding proteins (RBPs) and their associated RNAs. From left to right: (A) RIP (RNA immunoprecipitation): Cells are lysed, and the RBP is immunoprecipitated along with its bound RNAs under native or formaldehyde‐crosslinked conditions. Co‐precipitated RNAs are identified by RT‐qPCR or RNA‐seq. RIP may capture indirect associations when formaldehyde is applied as a crosslinking agent. (B) CLIP (CrossLinking and ImmunoPrecipitation): Cells are UV‐irradiated to covalently crosslink RBPs to directly bound RNAs. After immunoprecipitation, partial RNA digestion, and adapter ligation, high‐throughput sequencing reveals precise RBP binding sites at nucleotide resolution, minimizing background from indirect interactions. (C) TRIBE (targets of RNA‐binding proteins identified by editing): The RBP is fused to the catalytic domain of ADAR (adenosine deaminase acting on RNA), which edits adenosines (A) to inosines (I) near the RBP binding sites in vivo. A → I edits are detected as A → G mismatches in RNA‐seq data, providing transcriptome‐wide maps of RBP‐RNA interactions without requiring immunoprecipitation or crosslinking. (D) STAMP (surveying targets by APOBEC‐mediated profiling): Similar to TRIBE, STAMP fuses the RBP to APOBEC1, a cytidine deaminase that converts cytosines (C) to uracils (U) near RBP binding sites. Detection of C → U conversions in RNA‐seq data enables mapping of RBP‐RNA interactions with high sensitivity, though it remains unexplored in plant systems. (E) APEX2 (ascorbate peroxidase‐mediated RNA proximity labeling): The RBP is fused to the engineered peroxidase APEX2, which catalyzes biotinylation of nearby RNAs in the presence of biotin‐phenol and H_2_O_2_. Biotinylated RNAs are isolated with streptavidin and identified by sequencing, enabling spatial and temporal resolution of RBP‐associated RNAs in living cells. (F) Poly(U) polymerase (PUP)‐tagging: The *C. elegans* terminal uridylyltransferase PUP‐2 is fused to the RBP of interest, leading to the addition of uridines to the 3′ end of bound RNAs. These poly(U) tails can be used as molecular tags for selective RNA identification. This method functions independently of specific nucleotide editing or crosslinking and is suitable for identifying direct targets.

AS is regulated by RNA‐binding proteins (RBPs). Over 800 RBPs were identified in Arabidopsis, rice, and maize, reflecting the complexity of post‐transcriptional control (Silverman et al. 2013). To uncover which and how RBPs mediate AS, researchers apply either protein‐centric or RNA‐centric approaches to identify RNA targets of specific RBPs or the proteins interacting with a given transcript. These methods are broadly classified into in vitro and in vivo: In vitro assays use recombinant proteins or synthetic RNAs in pull‐down systems, while in vivo techniques often rely on epitope‐tagged RBPs in transgenic plants. Method choice depends on factors such as tissue specificity, transformation strategy, and antibody availability. An overview of each approach is provided in Figures 2 and 3, whereas in Table S2, for each method, the essential controls and selected validation methods are shown.

### 4.1 In Vitro Methods That Use RNA as a Bait

To identify proteins that bind to a specific RNA of interest, several in vitro methods have been developed, commonly referred to as RNA affinity purification or RNA pulldown assays. These methods begin with the in vitro transcription of the RNA bait, which is then incubated with native protein extracts, typically derived from a relevant plant tissue. RNA‐protein complexes are isolated, whereas unbound proteins are washed away. Bound proteins are identified via mass spectrometry (MS) or Western blotting, provided that suitable antibodies are available (Figure 2A). This strategy has been successfully applied in various plant systems (Seo and Chua 2019; Zhou et al. 2021; Reichel et al. 2024). The RNA may also be tagged with affinity labels such as biotin or desthiobiotin, enabling its capture using streptavidin‐coated magnetic beads (Figure 2B; Panda et al. 2016; Reichel et al. 2024). Alternative tagging strategies include the use of aptamer‐tagged RNAs that bind specific proteins or oligonucleotide‐based approaches using biotinylated DNA or locked nucleic acid (LNA) probes complementary to the target RNA and immobilized using carboxylated magnetic beads or biotinylated and enriched by streptavidin beads (Srisawat and Engelke 2001; Li and Altman 2002; Zheng et al. 2016). Gemmill et al. (2020) provide a comprehensive overview of RNA labeling chemistries, their technical advantages, and potential pitfalls.

Although advancements in MS have improved sensitivity, they have also increased background noise from non‐specific protein binding (Chen and Gingras 2007). To improve specificity, tandem affinity purification strategies such as TRAP‐MS (tandem RNA affinity purification followed by MS) have been developed. TRAP‐MS uses two sequential affinity tags to purify RNA‐protein complexes in two steps, thereby substantially reducing non‐specific background while increasing confidence in identified interactions (Li 2011; Krause and Simmonds 2004). However, this approach may compromise the detection of weak or transient RNA–protein interactions, which may still be captured by simpler AP‐MS (affinity purification followed by MS) protocols (Oeffinger 2012).

To balance sensitivity and specificity, an RNase‐assisted AP‐MS method that reduces bead‐associated background binding has been developed (Michlewski and Cáceres 2010). In this setup, following complex formation and washing, elution is achieved by an RNase treatment. Only proteins directly bound to RNA are released into solution, while bead‐bound contaminants remain attached (Michlewski and Cáceres 2010). Though validated in mammalian systems with microRNAs such as pri‐let‐7a‐1 and pri‐miR‐101‐1, this method has not yet been tested in plant systems (Swiatkowska et al. 2020; Szpotkowska et al. 2022). Another high‐throughput strategy is the use of protein microarrays. Here, fluorescently labeled RNA is hybridized to protein arrays, and binding events are detected by fluorescence scanning. This method enables rapid identification of candidate RBPs and offers high sensitivity, particularly for low‐abundance proteins (Siprashvili et al. 2012). With the emergence of plant‐specific protein arrays, this approach may become an efficient and scalable alternative for studying RNA–protein interactions in plants.

### 4.2 In Vivo Methods That Use RNA as a Bait

In vivo approaches, such as RNA interactome capture (RIC), preserve native RNA structure, localization, and modifications, enabling identification of protein partners in their physiological context. A common strategy involves crosslinking of the tissue. This preserves RNA–protein interactions during the purification procedure. After cell lysis, biotinylated antisense oligonucleotides hybridize to the endogenous target RNAs, followed by enrichment of crosslinked RNA‐protein complexes via streptavidin beads (Figure 2C). This was pioneered by Chu et al. (2011) using ChIRP‐MS (chromatin isolation by RNA purification‐MS), which allowed the recovery of proteins associated with non‐coding RNAs HOTAIR (HOX transcript antisense RNA) and TERC (telomerase RNA component) from human cell cultures. Capture Hybridization Analysis of RNA Target methods coupled with MS (CHART‐MS) employs fewer oligos and RNase H‐mediated elution offers enhanced RNA‐RBP specificity (Simon et al. 2011). CHART‐MS has been applied so far only to human cell lines, and its application to plant systems is still lacking.

Tissue crosslinking, typically done with formaldehyde or glutaraldehyde, helps preserve RNA–protein interactions during purification but can also trap indirect interactors by crosslinking all macromolecules (Srinivasan et al. 2002). Ultraviolet (UV) crosslinking induces covalent bonds primarily between RNA and proteins, minimizing non‐specific or indirect interactions that are not part of the true RNA–protein complexes of interest (Chodosh 1996). UV crosslinking is irreversible and may result in low recovery, but this can be improved with more input material, better probe design, and sensitive MS. Generally, it should be noted that UV crosslinking is less efficient than chemical crosslinking in plant systems due to their high content of chlorophylls, carotenoids, flavonoids, and other phenolic compounds that absorb UV light. In contrast, chemical crosslinkers must overcome the barrier of the cell wall and, depending on the tissue, additional extracellular layers such as the cuticle. An overview of different crosslinking techniques, their advantages and disadvantages, as well as their considerations in plant systems, is provided by Van Ende et al. (2020).

The PIP‐seq (protein interaction profile sequencing) method provides a transcriptome‐wide view of RNA–protein interactions and RNA secondary structures in vivo (Silverman et al. 2014). It combines protein crosslinking (e.g., by UV light) with RNase digestion and next‐generation sequencing. Bound regions are protected from nuclease activity, allowing mapping of protein footprints and RNA structures at high resolution as previously shown for Arabidopsis root cells (Foley et al. 2017). PIP‐seq thus bridges structural and interaction profiling, making it a powerful tool for uncovering regulatory features of the plant transcriptome. LNA oligonucleotides improve specificity and stability in RIC by forming tighter RNA hybrids and resisting nucleases (Obika et al. 1997; Wahlestedt et al. 2000; Rogell et al. 2017; Reichel et al. 2024).

Proximity labeling technologies offer an added layer of precision by tagging proteins near specific RNAs in vivo. RNA‐BioID uses MS2‐tagged RNAs and MCP‐BirA* fusions to biotinylate nearby proteins, while RaPID employs λN‐BirA* to target BoxB‐tagged RNAs (Ramanathan et al. 2018; Mukherjee et al. 2019). In both methods, biotinylated proteins are enriched via streptavidin purification and identified by MS, requiring co‐expression of the tagged RNA and fusion protein components (Figure 2D). To the best of our knowledge, RNA‐BioID and RaPID have not yet been applied to plant systems. Their implementation would require either a transient or a stable expression system.

Clustered regularly interspaced short palindromic repeats (CRISPR)‐based proximity labeling is a major advance for mapping RNA–protein interactions without modifying the RNA itself. These methods use catalytically inactive/dead Cas13 (dCas13) fused to biotin ligases, which, guided by a gRNA, bind specific RNAs and biotinylate nearby proteins. Labeled proteins are then purified with streptavidin beads and identified by MS (Figure 2E) (Abudayyeh et al. 2016; Burmistrz et al. 2020). Examples include the CRISPR‐assisted RNA–protein interaction detection method (CARPID; Yi et al. 2020), Ribonucleic acid proximity protein labeling (RiboPro; Lin et al. 2020), and the CRISPR‐based RNA‐united interacting system (CRIUS; Zhang et al. 2020). These approaches enable in vivo mapping of RNA–protein interactions with high spatial and temporal resolution. Their efficacy depends on gRNA design targeting unstructured single‐stranded RNA regions (Bandaru et al. 2020). Though mainly applied in animal systems, these tools are highly promising for plant research. They avoid RNA tagging, which is technically challenging in plants, and are suitable for studying native RNAs in diverse tissues or stress conditions. As mentioned for previous proximity‐labeling methods, their implementation in plants would require either a transient or a stable expression system. Using tissue‐specific or inducible promoters could facilitate interaction profiling in crops or non‐model species with limited genomic resources.

Yeast‐based systems such as the yeast three‐hybrid (Y3H) assay (Cho and Hannapel 2018) enable the identification of RBPs by screening cDNA or RBP libraries. In this setup, the GAL4 DNA‐binding domain is fused to the MS2 coat protein (MCP) and co‐expressed with an MS2‐tagged RNA of interest. Yeast cells are transformed with candidate RBPs fused to the GAL4 activation domain. If an RBP binds the RNA, it brings the DNA‐binding domain and activation domain into proximity, activating a reporter gene, typically allowing growth on selective media. This system is modular, does not require prior knowledge of potential interactors, and is suitable for identifying plant RBPs by using expression libraries. It offers a relatively simple and scalable platform to uncover novel RNA–protein interactions. Attention should be paid to the fact that yeast cells are not identical to plant cells and that, therefore, a direct transfer of conclusions is not possible.

Together, these in vivo strategies provide powerful insights into dynamic RNA‐protein networks in plant systems under native physiological conditions.

### 4.3 In Vitro Methods Using an RNA‐Binding Protein as a Bait

One of the oldest methods to identify in vitro RNA motifs that are bound by a protein of interest is SELEX (Systematic Evolution of Ligands by EXponential enrichment; Tuerk and Gold 1990). SELEX relies on the binding of specific RNA sequences from a random pool of short RNAs to a purified protein of interest. Bound RNAs are enriched by reverse transcription and PCR amplification. This cycle is repeated multiple times, and the final pool of sequences is sequenced. The results are artificial sequences that can be analyzed to identify a preferred enriched binding motif. Though widely used, including for plant RBPs (Zhang and Muench 2015), SELEX can introduce biases due to PCR and the artificial nature of the RNA pool. To address these limitations, RNAcompete was developed (Ray et al. 2017). It uses a custom‐designed RNA pool in a single binding reaction, followed by detection via microarrays or sequencing. Although faster and less biased, it may miss motifs not represented in the designed pool and is less stringent than SELEX (Ray et al. 2013; Köster et al. 2025).

RNA affinity purification sequencing (RAP‐seq) employs fragmented, endogenous RNA directly extracted from tissues (Atanasoai et al. 2021). The purified native RNA is incubated with an immobilized protein of interest, and bound transcripts are identified via RNA‐seq. Unlike SELEX and RNAcompete, RAP‐seq retains physiological RNA features such as post‐transcriptional modifications, offering a more biologically relevant view of RNA–protein associations. However, its application to plant RBPs remains unexplored.

Similarly, SNAAP (specific nucleic acids associated with proteins) replicates native intracellular conditions by incubating immobilized RBPs with whole‐cell extracts (Trifillis et al. 1999). Following enrichment, RNA‐seq identifies both directly and indirectly associated RNAs. The native extract preserves not only RNA modifications and structures but also co‐existing protein complexes, more accurately reflecting in vivo interactions. This setup can reveal RNA targets missed by synthetic systems, although it may also capture indirect associations. SNAAP has been successfully used in Arabidopsis (Cheng et al. 2003), highlighting its versatility across species.

### 4.4 In Vivo Methods Using an RNA‐Binding Protein as a Bait

Although informative, in vitro methods often fail to fully recapitulate the complexity of the cellular environment. In contrast, in vivo approaches preserve the native molecular environment, enabling detection of physiological RNA–protein interactions and capturing their dynamic nature, especially under various biotic and abiotic stimuli.

RNA immunoprecipitation (RIP) is one of the most established techniques. RIP involves cell lysis, followed by the immunoprecipitation of the RBP along with bound RNAs, and identification of the RNAs via RT‐qPCR or RNA‐seq (Figure 3A; Juntawong et al. 2013). RIP can be performed under native conditions or following formaldehyde crosslinking, which is commonly used prior to lysis to stabilize transient RNA–protein interactions (Terzi and Simpson 2009).

RIP has been widely adopted in plant RNA splicing research, including Arabidopsis (Bazin et al. 2018) and tomato (Rosenkranz et al. 2024). Transgenic lines expressing RBPs fused with protein tags such as GFP or FLAG may be used if no specific antibodies are available. RIP has also been adapted for use in transiently transformed tissues and protoplast systems, enabling rapid analysis without the need for stable transgenic lines (Marmisolle et al. 2018; Zhou et al. 2021). RIP material can be used either for PCR when the target is known or for RNA‐seq to reveal associated RNAs. Formaldehyde crosslinking can increase the risk of false positives, especially when appropriate negative controls are lacking. For instance, when studying a nuclear protein tagged with GFP, an ideal negative control would be GFP fused to a nuclear localization signal (NLS), ensuring comparable nuclear localization without specific RNA binding. Moreover, maintaining similar expression levels between the protein of interest and the control is critical, as differences in protein abundance can skew results. Overexpression of a protein of interest can further introduce artifacts by promoting non‐physiological interactions. These controls apply for all methods using RBP as bait.

Formaldehyde crosslinking may cause artifacts and capture indirect interactions due to the crosslinking of protein–protein interactions; therefore, in a non‐native RIP assay it will provide information on associated and not only directly bound RNAs (Mili and Steitz 2004). Direct protein–RNA interaction can be probed by CLIP (CrossLinking and ImmunoPrecipitation), which uses UV irradiation to covalently link proteins to RNAs, reducing indirect background (Figure 3B; Ule et al. 2003). CLIP and its variants (e.g., individual‐nucleotide resolution CLIP (iCLIP), enhanced CLIP (eCLIP)) have so far been applied in Arabidopsis (Zhang et al. 2015; Meyer et al. 2017; Lewinski, Brüggemann, et al. 2024) and tomato (Rosenkranz et al. 2024).

Recently, a combination of protein proximity‐labeling and RIP enabled the identification of the RNA composition of processing bodies in Arabidopsis (Liu et al. 2024). Given that many splicing factors localize to biomolecular condensates, such integrative approaches could provide more comprehensive insights into their functional roles.

To reduce background further, UV crosslinking and an affinity purification method (uvCLAP) combine UV crosslinking with a tandem purification system using a 3FHBH (3xFLAG–His_6_–Biotinylation sequence–His_6_) tag (Maticzka et al. 2018), although this has not been applied in plants yet. Another advanced technique is TRIBE (targets of RNA‐binding proteins identified by editing), where the RBP is fused to ADARcd (ADENOSINE DEAMINASE ACTING on RNA catalytic domain), an RNA‐editing enzyme that converts adenosine to inosine at binding sites (McMahon et al. 2016). These edits are detected as A → G substitutions in RNA‐seq (Figure 3C). An advanced version of TRIBE, called HyperTRIBE, increases editing efficiency using a catalytic domain of ADAR carrying an E488Q mutation (Rahman et al. 2018). TRIBE was recently applied to OsDRB1 in rice (Yin et al. 2023). Another technique using the same principle as TRIBE called STAMP (surveying targets by APOBEC‐mediated profiling) uses cytosine deaminase APOBEC1 to convert C to U (Figure 3D; Brannan et al. 2021). Loeser et al. (2024), compared the activity of several RNA‐editing enzymes and confirmed the activity of ADAR, while APOBEC enzymes showed no editing activity in *N. benthamiana* (Loeser et al. 2024). These editing‐based methods avoid immunoprecipitation but depend on the presence of editable nucleotides (adenosines or cytosines) near RBP binding sites and suitable RNA structure (Macbeth et al. 2005; Eggington et al. 2011).

A related approach is polyuridylation tagging, in which the *Caenorhabditis elegans* polymerase PUP‐2 is fused to the RBP, adding uridines to 3′ ends of bound RNAs (Lapointe et al. 2017). Although this method lacks high‐resolution binding site mapping, it avoids the need for specific nucleotide editing. Notably, it was shown in Arabidopsis that uridylation of mRNAs has a dual role in mRNA degradation as well as stabilization, making further case‐specific analyses essential (Scheer et al. 2016). Further, proximity labeling with the engineered ascorbate peroxidase APEX2 has recently shown promising results for direct RNA tagging. APEX2 biotinylates guanines on RNAs near the RBP of interest, allowing identification via streptavidin pull‐down and sequencing (Figure 3E; Zhou et al. 2019). In plant systems, the RNA labeling activity of APEX2 has only been assessed by Liu et al. (2024), and no labeling was observed, indicating that further adjustments and optimization are needed.

In summary, a diverse suite of in vivo techniques, from RIP and CLIP to TRIBE and proximity labeling, is now available or being adapted for plant research. These tools vary in resolution, efficiency, and technical complexity. IP‐based methods like iCLIP and RIP are highly effective at capturing RNAs bound to specific proteins without relying on a transgenic system, assuming suitable antibodies are available. However, these methods are limited in their crosslinking capabilities and IP efficiency. On the other hand, labeling methods such as TRIBE, STAMP, proximity labeling, and PUP‐tagging eliminate the need for crosslinking. However, they require a transgenic system and the optimal activity of the enzyme involved. Collectively they offer unprecedented insights into the spatial and temporal dynamics of RNA–protein interactions within their natural cellular context.

## 5 Validation of Splicing Factor Function and Using Minigene Assays

Minigene assays are versatile tools to investigate how pre‐mRNA splicing is regulated in specific gene regions. A minigene is a simplified gene construct that includes essential exonic and intronic sequences involved in splicing. These regions are cloned into reporter plasmids, enabling in vivo analysis of *cis*‐regulatory elements and *trans*‐acting splicing factors (Cooper 2005; Sharma et al. 2014; Tamayo et al. 2023).

**FIGURE 4:**
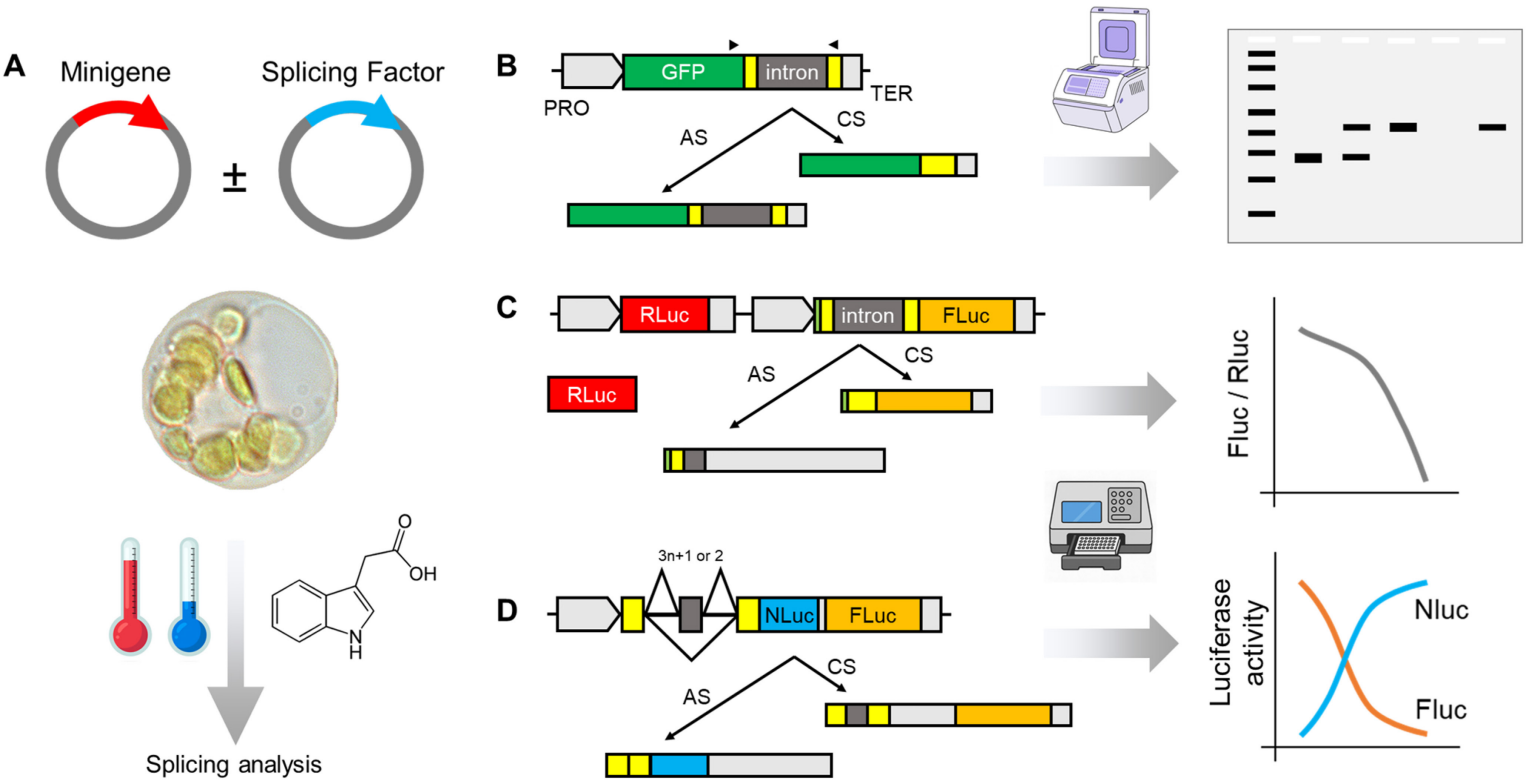
Analysis of splicing using minigene assays. (A) General workflow of a minigene assay. Plasmids encoding the minigene construct and, optionally, a splicing factor of interest are co‐transfected into a transient expression system (e.g., protoplasts). Splicing analysis is performed under basal or treatment conditions (e.g., abiotic stress or small molecules). (B) RT‐PCR‐based minigene assay. A genomic fragment containing an intron and flanking truncated exons is cloned in‐frame upstream of a green fluorescent protein (GFP) coding sequence. After transfection, RNA is extracted, reverse‐transcribed, and analyzed by RT‐PCR using specific primers (arrowheads). One primer anneals within GFP to avoid amplification of the endogenous gene. Splicing isoforms are resolved by agarose or capillary electrophoresis. (C) Single‐reporter luciferase minigene assay. The minigene is cloned upstream of a luciferase gene (e.g., Firefly [FLuc]), where splicing removes a premature termination codon to allow translation. An independent Renilla luciferase (RLuc) gene serves as a normalization control. (D) Dual‐reporter luciferase minigene assay. A cassette of three exons and two introns is cloned upstream of two out‐of‐frame tandem luciferase reporters (e.g., NanoLuc [NLuc] and Firefly [FLuc]). Distinct splicing outcomes (e.g., exon skipping vs. inclusion) restore the reading frame of only one luciferase, enabling quantitative assessment of isoform ratios based on differential reporter activity. AS, alternatively spliced; CS, constitutively spliced; PRO, promoter; TER, terminator.

Minigenes are typically expressed under a constitutive promoter, though condition‐specific promoters (e.g., heat‐inducible promoters of heat‐shock proteins) can be used to examine stress‐responsive splicing (Figure 4A). Constructs are introduced into transient expression systems, such as protoplasts or tobacco leaves, often co‐transfected with splicing regulators (Tyurin et al. 2020). Mutations in *cis*‐elements introduced via site‐directed mutagenesis can help determine their role in AS.

Minigene assays enable rapid screening of binary AS events, such as IR or exon skipping, using RT‐PCR (Figure 4B) or various reporter systems (e.g., GFP or Firefly luciferase; Figure 4C). In single‐reporter constructs, AS often introduces premature termination codons, leading to reduced expression of the fused minigene with the GFP or Firefly luciferase reporter, which can be normalized against a co‐expressed transcriptional control such as Renilla luciferase (Stauffer et al. 2010; Li et al. 2021; Rosenkranz et al. 2024). Dual‐reporter designs incorporate different luciferases (e.g., NanoLuc and Firefly) at either end of the minigene (Figure 4D), allowing simultaneous detection of splice isoforms (Li et al. 2021; Best et al. 2024). Constitutive splicing results in the generation of a single isoform corresponding to the default reporter, whereas AS generates additional isoforms that are represented by the second reporter in this system. This setup permits quantification of splicing events and normalization to a transcriptional control, enhancing sensitivity and reliability in functional splicing assays.

Although they cannot resolve complex splicing patterns, minigene assays provide a scalable platform to identify key splicing regulators. They are useful for screening splicing factors or even small‐molecule libraries that modulate specific AS events.

## 6 Biophysical and Biochemical Approaches to Study Protein–RNA Interactions

After identifying candidate RBPs and their RNA targets, in vitro methods are used for mechanistic validation. Techniques like electrophoretic mobility shift assays (EMSA), isothermal titration calorimetry (ITC), and fluorescence‐based assays can determine binding affinity, specificity, and stoichiometry, providing key insights into RNA–protein interactions in splicing and broader RNA regulation and further validate results obtained by global approaches mentioned in section 4.

### 6.1 Electrophoretic Mobility Shift Assay (EMSA)

**FIGURE 5:**
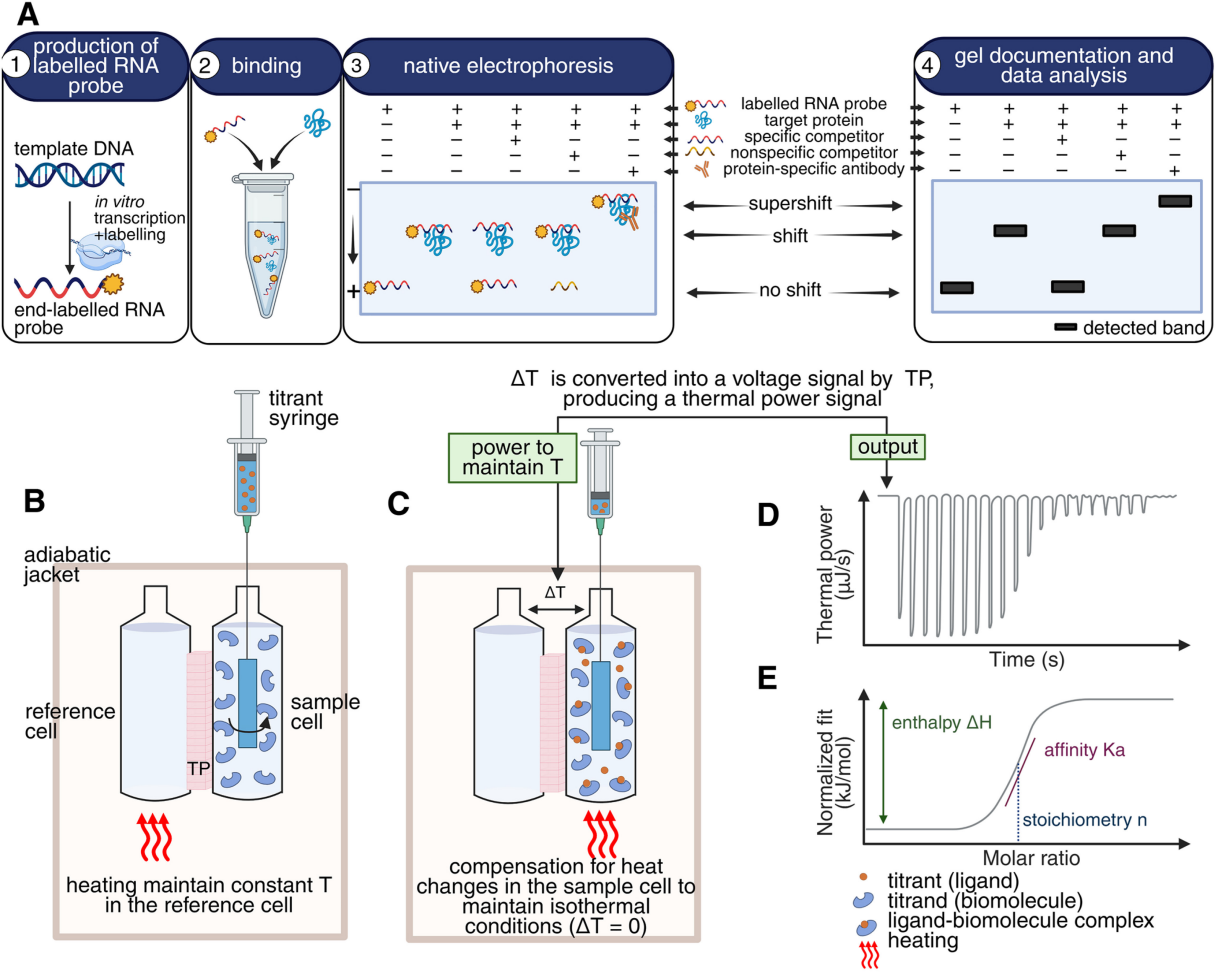
Biophysical and biochemical approaches to study protein–RNA interactions. RNA electrophoretic mobility shift assay (RNA EMSA) and isothermal titration calorimetry (ITC). (A) The EMSA analysis consists of four steps: (i) synthesis of target nucleic acid fragment and labeling with radioactive, fluorescent, or biotin tags usually from one of the terminal ends to reduce the impact of the labeling on interaction; (ii) incubation of labeled RNA probe with a protein sample and controls with specific and/or nonspecific competitors, (iii) native polyacrylamide gel electrophoresis, and (iv) gel documentation depending on selected labeling. (B) Example of an ITC instrument with cylindrical cells. Reference and sample cells are fixed in an adiabatic jacket, maintaining constant temperature and pressure. (C) The titrant is injected with the syringe gradually; upon interaction, the temperature changes in the sample cell, and the temperature difference (Δ*T*) between the reference and sample cells is converted into a voltage signal by the thermopile (TP), producing a thermal power signal. Typical ITC graphs: (D) Thermogram of exothermic reaction showing thermal power plotted as a function of time of titration, and (E) binding isotherm showing peaks from the thermogram (D) plotted as a function of the ratio between titrant and titrant concentration (Salim and Feig 2009; Saponaro 2018; Bastos et al. 2023; created with BioRender).

EMSA is used to detect and characterize direct interactions between proteins and nucleic acids (Rio 2014). It is based on the principle that an RNA or DNA molecule bound to a protein exhibits reduced electrophoretic mobility under non‐denaturing conditions, forming a distinct shifted band of higher molecular weight (Figure 5A; Hellman and Fried 2007; Shen and Fried 2012). A key advantage of EMSA is its low material requirement and cost, with native conditions preserving functional interactions. At the same time, it can also be used with crude extracts if the RBP is sufficiently expressed. When performed using varying concentrations of labeled RNA probe and constant protein levels, EMSA can be used to determine the dissociation constants (*K*_*d*_) for RNA–protein interactions (Adams and Fried 2007). Labeling of the RNA probe is recommended for detection. Radioactive labeling provides the highest sensitivity (England and Uhlenbeck 1978) but raises safety and disposal issues. Alternatives like biotinylation (Daras et al. 2019) or fluorescent dyes (Lamichhane et al. 2010; Hsieh et al. 2016) are safer to handle and the protocol will be faster to implement, although potentially less sensitive or structurally disruptive. EMSA specificity is confirmed using specific and non‐specific competitor RNAs such as unlabeled (cold) RNA, or non‐specific competitors such as tRNA or heparin (Carey et al. 2013; Wang et al. 2022), whereas supershift assays with RBP‐targeting antibodies can verify protein identity (He et al. 2024).

Despite its strengths, EMSA does not provide information on the exact binding site or structural dynamics of the interaction. Weak complexes may dissociate during electrophoresis, especially if running conditions are not optimized. Furthermore, bulky probes or suboptimal labeling can impair complex formation or alter migration patterns. EMSA is well suited for early‐stage validation of RBP–RNA interactions identified by in vivo approaches. It requires only modest prior knowledge of the RBP‐RNA pair being tested, mainly the RNA sequence to be tested, and is a valuable tool for quantifying relative binding affinities or testing the effects of mutations in the RNA sequence on binding affinities to the tested RBP.

### 6.2 Isothermal Titration Calorimetry

ITC directly measures the heat released or absorbed during biomolecular interactions, offering label‐free quantification of binding parameters such as the association constant (*K*_*a*_), stoichiometry (*n*), enthalpy (Δ*H*), and entropy (Δ*S*; Feig 2009; Falconer and Collins 2011; Bastos et al. 2023). In a typical ITC experiment, an RNA fragment is titrated into a sample cell of an ITC unit containing the purified RBP (Figure 5A,B). The instrument detects small temperature changes caused by binding between the RNA fragment and the RBP, generating a thermogram that reflects the kinetics and thermodynamics of the interaction (Figure 5C–E; Gilbert and Batey 2009). ITC is best used as a confirmatory method when prior evidence suggests a direct RBP–RNA interaction, providing mechanistic insight rather than serving as a screening tool.

ITC preserves protein and RNA in native solution conditions, avoiding artifacts from labeling or immobilization (Saponaro 2018; Glöckner and Klebe 2021). However, ITC is less suitable for very weak or ultra‐tight binding interactions unless adapted protocols are applied (Velazquez‐Campoy and Freire 2006). Accurate results demand that both RNA and protein be in identical buffer conditions to avoid nonspecific thermal effects (Bastos et al. 2023). Although assay setup is straightforward, the interpretation of complex binding models may require advanced fitting approaches.

### 6.3 Nuclear Magnetic Resonance (NMR) Spectroscopy

NMR spectroscopy can be used to determine interaction interfaces, including the identification of specific amino acid residues involved in RNA binding. For these experiments, isotopically labeled protein samples and a short, highly purified RNA fragment are required. By recording a series of two‐dimensional ^1^H–^15^N heteronuclear single quantum coherence (HSQC) spectra with varying RNA concentrations, one can monitor chemical shift perturbations (CSPs) of individual amino acid residues. These CSPs can be used to map the RNA‐binding surface of the protein and can be further analyzed to estimate *K*_*a*_ (Schlundt et al. 2017; Lewinski, Steffen, et al. 2024).

NMR can be integrated with other structural techniques such as small angle X‐ray and/or neutron scattering (SAXS/SANS), electron paramagnetic resonance (EPR), and Förster resonance energy transfer (FRET). Together, these techniques synergistically enhance the understanding of the intricate arrangements, overall shapes, and dynamic behaviors of molecules. However, their application is limited by high sample requirements, size constraints, and spectral complexity, particularly when dealing with large or highly dynamic RNP complexes (Schlundt et al. 2017).

### 6.4 Fluorescence‐Based Techniques

Fluorescence‐based methods provide highly sensitive, real‐time measurements of RNA–protein interactions and are often used when ITC or EMSA are impractical. These include FRET, fluorescence anisotropy, and microscale thermophoresis (MST). The principle of FRET relies on the non‐radiative transfer of energy between the two fluorophores, a donor and an acceptor, when they are in close proximity (1–10 nm). Both molecules are tagged/labeled with compatible fluorophores, allowing the detection of binding events or conformational changes (Lamichhane et al. 2010; Shen and Fried 2012). Fluorescent RNA probes can be synthesized or labeled at the 5′ or 3′ ends using dyes such as FAM or Cy3 (Eguchi 2007; Brown et al. 2020). This approach eliminates the need for hazardous isotopes, provides dynamic binding data, and allows kinetic measurements. Some techniques, like FRET, are particularly informative for splicing factor studies, as they detect structural rearrangements within the RNA upon protein binding, and are useful for understanding regulatory mechanisms.

However, fluorescence‐based methods require specialized equipment and careful design. Fluorophores must be positioned not to perturb the native structure, and signal interference from sample autofluorescence or quenching must be controlled. The labeling complexity and optimization efforts are higher in dual‐label systems like FRET than in single‐label formats. These techniques are most powerful when sufficient prior knowledge exists to guide probe design and when the goal is to dissect interaction dynamics or screen multiple variants with high sensitivity and throughput.

## 7 Future Perspectives

Machine learning (ML) and artificial intelligence (AI) complement experimental and computational techniques to support RNA‐seq which can suffer from limitations in SJ coverage and low‐abundance isoform detection. ML‐based tools and AI models can enhance AS studies by enabling the prediction of splicing patterns, identifying *cis*‐regulatory elements, and prioritizing candidate splicing factors. In plant research, the use of AI and ML is not yet as advanced as in mammals. ML models such as DeepASmRNA classify AS event types even in species lacking a reference genome, greatly expanding AS exploration in non‐model organisms (Cao et al. 2022). Deep learning can also automate complex feature extraction from raw sequence data, improving the prediction accuracy of splice sites and branch points without manual annotation (Chao et al. 2024). AI‐based structure models like AlphaFold3 hold promise for predicting RNA–protein interactions, but current limitations, especially for non‐canonical contacts, mean predictions should be used cautiously. Integrating AI models with experimental data and docking tools offers a practical path forward until training datasets improve (Hennig 2025).

Furthermore, the current tissue‐focused analysis of AS often masks cell‐type‐specific splicing events due to the averaging effect across heterogeneous cell populations. Single‐cell RNA sequencing (scRNA‐seq) overcomes this limitation by enabling transcriptomic profiling at cellular resolution, allowing the identification of AS events unique to specific cell types or developmental stages. Integrating scRNA‐seq into splicing research is thus essential to uncover the whole landscape of splicing regulation in complex plant tissues and to resolve spatial and temporal patterns of AS that remain invisible in bulk RNA‐seq datasets. However, AS remains largely underexplored at single‐cell levels, mainly due to low sequencing depth (Nobori 2025). Most available methods and pipelines have been developed for animal systems and are not optimized for plant‐specific AS features, such as the predominance of IR. Tools like BRIE and VALERIE, among others, show promise in analyzing AS in scRNA‐seq datasets, but their application in plants is still limited as they are not adapted to plant‐specific features (Wen et al. 2020; Huang and Sanguinetti 2021).

**FIGURE 6:**
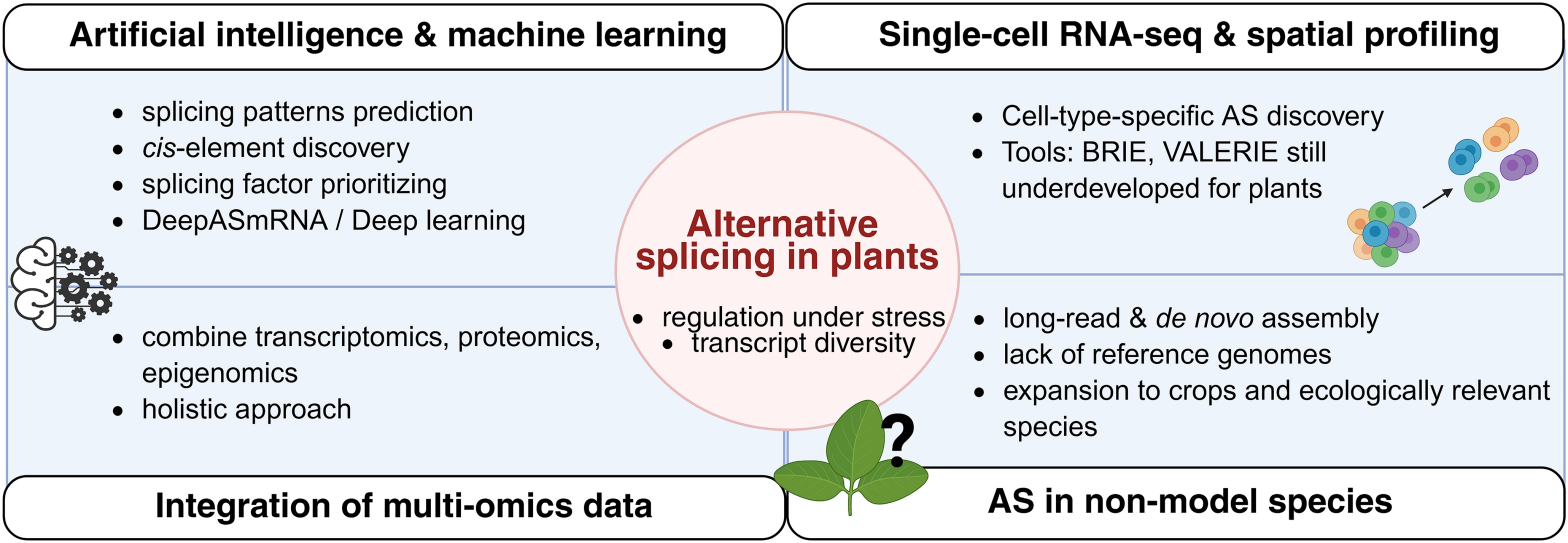
Emerging directions and challenges in plant alternative splicing (AS) research. Ongoing developments in plant molecular biology highlight several promising directions for advancing our understanding of AS regulation. Artificial intelligence and machine learning approaches are promising to increase efficiency in predicting splicing patterns, identifying *cis*‐regulatory elements, and prioritizing splicing factors. Integrating multi‐omics datasets enables a more holistic understanding of AS regulation and its role in stress adaptation. Single‐cell RNA sequencing and spatial transcriptomics offer cell‐type‐specific insights but remain underdeveloped for plants, with tools such as BRIE and VALERIE still requiring optimization. The adoption of long‐read and de novo assembly methods can overcome the limitations of incomplete reference genomes, facilitating AS studies in crops and ecologically relevant non‐model species (created with BioRender).

Moreover, expanding AS research beyond model species is crucial. Many agriculturally important crops and ecologically relevant species lack reference genomes or standardized tools, yet they exhibit extensive AS variations critical for stress adaptation and development. Advances in long‐read sequencing, de novo transcriptome assembly, and ML–based predictions enable high‐resolution splicing studies in non‐model systems. This shift will allow researchers to translate mechanistic insights from model plants to crop species and identify splicing events and regulators that can be targeted to improve stress tolerance, yield, and resilience under changing climates. Thus, integrating multi‐omics data and expanding to non‐model species will be essential steps toward a holistic and application‐oriented understanding of AS in plants (Figure 6).

## Author Contributions

**Stavros Vraggalas:** writing – review and editing, writing – original draft, and conceptualization. **Oussama Guennich:** writing – review and editing, writing – original draft, funding acquisition. **Boushra Shalha:** writing – review and editing, writing – original draft. **Christos Bazakos:** writing – review and editing. **Hélène S. Robert:** writing – review and editing, funding acquisition. **Olha Lakhneko:** writing – review and editing, writing – original draft, visualization, funding acquisition, and conceptualization. **Sotirios Fragkostefanakis:** writing – review and editing, writing – original draft, visualization, funding acquisition, and conceptualization.

## Supporting information


**Table S1:** Tools commonly used for each step in long‐read AS analysis pipelines.
**Table S2:** Overview of experimental approaches to study RNA–protein interactions.

## Data Availability

Data sharing is not applicable to this article as no new data were created or analyzed in this study.
