# Supplementary material for: Methods for Analyzing Alternative Splicing and Its Regulation in Plants: From Gene‐Specific Approaches to Transcriptome‐Wide Studies
Source: Physiol Plant. 2025 Nov 18;177(6):e70639. doi: 10.1111/ppl.70639 (PMC12626760; doi:10.1111/ppl.70639)
Supplement: Supplementary file 1 — Table S1: Tools commonly used for each step in long‐read AS analysis pipelines. Table S2: Overview of experimental approaches to study RNA–protein interactions. [file PPL-177-e70639-s001.pdf]

Methods for analyzing alternative splicing and its regulation in plants: From gene-specific approaches to transcriptome-wide studies

Stavros Vraggalas[1‡], Oussama Guennich[2,3,‡], Boushra Shalha[1,‡], Christos Bazakos[4], Hélène S. Robert[2], Olha Lakhneko[5, *], Sotirios Fragkostefanakis[1, *]

[1] Molecular and Cell Biology of Plants, Institute of Molecular Biosciences, Goethe University Frankfurt, D-60438, Frankfurt am Main, Germany

[2] Mendel Center for Plant Genomics and Proteomics, CEITEC MU—Central European Institute of Technology, Masaryk University, Brno, 625 00, Czech Republic

[3] National Centre for Biomolecular Research, Faculty of Science, Masaryk University, Brno, 625 00, Czech Republic

[4] Institute of Plant Breeding and Genetic Resources, ELGO DIMITRA, Thermi, Greece

[5] Institute of Plant Genetics and Biotechnology, Plant Science and Biodiversity Centre, Slovak Academy of Sciences, Akademická 2, 950 07 Nitra, Slovakia

[‡]Equal contribution

*Corresponding authors: SF: fragkost@bio.uni-frankfurt.de; OL: olha.lakhneko@savba.sk

Table S1. Tools commonly used for each step in long-read alternative splicing analysis pipelines.

Pipeline Step	Software Examples	References
Basecalling	Guppy, SMRT Link	Rhoads and Au 2015; Raghavachari and Garcia-Reyero 2018; Zhao et al. 2019
Adapter/Barcode Trimming	Porechop, Lima	Rhoads and Au 2015; Zhao et al. 2019
Quality Filtering	Filtlong, Chimeric Filter	Moldován et al. 2018; Zhao et al. 2019
Full-length Transcript Detection	Iso-Seq, Pychopper	Raghavachari and Garcia-Reyero 2018; Zhao et al. 2019
Error Correction	Proovread, LoRDEC, Canu	Hackl et al. 2014; Salmela and Rivals 2014; Koren et al. 2017
Clustering and Polishing	Iso-Seq Clustering, RATTLE	Zhao et al. 2019; de la Rubia et al. 2022
Read Mapping	minimap2, GMAP, STARlong	Wu and Watanabe 2005; Dobin et al. 2013; Li 2018
Transcript Annotation	cDNA Cupcake, TAMA	Wang et al. 2018; Zhang et al. 2019
Genome-free Isoform Reconstruction	Cogent	Tseng et al. 2016
Splice Junction Correction	Short-read-supported filtering	Li and Li 2019

Table S2. Overview of experimental approaches to study RNA-protein interactions.
This table summarizes the methods described in the review that are used to identify RNA–protein interactions, their general type, critical experimental controls, and suggested validation strategies. WT: Wild type – a not stable or transiently transformed plant tissue. SHAPE-MaP: Selective 2´-hydroxyl acylation analyzed by primer extension and mutational profiling. DMS-MaP: Dimethyl sulfide mutational profiling.

Method	Type	Essential Controls	Possible Validation Methods
**RNA pull-down / AP-MS**	*In vitro*, RNA as bait	- Unrelated/non-specific RNA<br>- IP using beads only	- Western blot for known RBPs<br>- Mass spectrometry reproducibility across replicates<br>- *In vitro* validation for specific interactions (e.g., EMSA)
**TRAP-MS (tandem affinity purification)**	*In vitro*, RNA as bait	- First-step pull-down only<br>- Mutated/deleted RNA motif<br>- IP using beads only	- Western blot for known RBPs<br>- Mass spectrometry reproducibility across replicates - *In vitro* validation for specific interactions (e.g., EMSA)
**RNase-assisted AP-MS**	*In vitro*, RNA as bait	- Elution without RNase treatment<br>- Mutated/deleted RNA motif<br>- IP using beads only	- Western blot for known RBPs<br>- Mass spectrometry reproducibility across replicates<br>- *In vitro* validation for specific interactions (e.g., EMSA)
**RNA–protein microarrays**	*In vitro*, RNA as bait	- Scrambled RNA probe<br>- Protein-free spots	- *In vitro* validation for specific interactions (e.g., EMSA)
**RIC (RNA interactome capture)**	*In vivo*, RNA as bait	- No-crosslink control<br>- Scrambled or non-specific probes<br>- IP using beads only	- Western blot for known RBPs<br>- Mass spectrometry reproducibility across replicates<br>- *In vitro* validation for specific interactions (e.g., EMSA)
**ChIRP-MS / CHART-MS**	*In vivo*, RNA as bait	- Scrambled or irrelevant probes<br>- Comparison of no-crosslinking, chemical crosslinking, and UV crosslinking<br>- IP using beads only<br>- Elution without RNase treatment	- Western blot for known RBPs<br>- Mass spectrometry reproducibility across replicates<br>- *In vitro* validation for specific interactions (e.g., EMSA)
**PIP-seq**	*In vivo*, RNA as bait	- No-crosslink control	- Compare with *in vivo* and *in vitro* structural probing methods, e.g., SHAPE-MaP and DMS-MaP<br>- Reproducibility across replicates
**RNA-BioID / RaPID**	*In vivo*, RNA as bait (proximity labeling)	- RNA without tag<br>- Different proximity labeling enzymes<br>- Expression of a proximity labeling enzyme without the λN peptide for RaPID or without MCP for RNA-BioID	- Western blot validation of biotinylated proteins<br>- Reproducibility across replicates
**CRISPR–dCas13 proximity labeling (CARPID,**	*In vivo*, RNA as bait (gRNA-guided)	- Non-targeting gRNA<br>- gRNA against unrelated RNA<br>- dCas13 alone	- Different gRNAs targeting unstructured vs. structured regions<br>- Reproducibility across replicates

**RiboPro, CRUIS)**			
**Yeast Three-Hybrid (Y3H)**	*In vivo* (yeast), RNA as bait	- Empty prey vector<br>- RNA without MS2 tag<br>- Mutated/deleted RNA motif<br>- Known non-binding RBP	- Validation of results *in vitro* and *in vivo* (in planta)<br>- Reproducibility across replicates
**SELEX**	*In vitro*, protein as bait	- PCR round without protein<br>- Without bait protein or with an RNA-binding–impaired mutant of the bait protein	- Validation of binding motifs with other in vitro methods (e.g., EMSA, ITC, etc.)<br>- Comparison with *in vivo* binding data for the bait protein (e.g., RIP, CLIP, etc.)
**RNAcompete**	*In vitro*, protein as bait	- PCR round without protein<br>- Mutated motif enrichment test<br>- Without bait protein	- Validation of binding motifs with other *in vitro* methods (e.g., EMSA, ITC, etc.)<br>- Comparison with *in vivo* binding data for the bait protein (e.g., RIP, CLIP, etc.)<br>- qRT-PCR for known targets as readout
**RAP-seq**	*In vitro*, protein as bait	- IP without the bait protein or with an RNA-binding–impaired mutant of the bait protein	- Validation of bound RNAs with *other in vitro* methods (e.g., EMSA, ITC, etc.)<br>- Comparison with *in vivo* binding data for the bait protein (e.g., RIP, CLIP, etc.)<br>- qRT-PCR for known targets as readout
**SNAAP**	*In vitro*, protein as bait	- IP without bait protein or with an RNA-binding–impaired mutant of the bait protein	- Validation of bound RNAs with other *in vitro* methods (e.g., EMSA, ITC, etc.)<br>- Comparison with *in vivo* binding data for the bait protein (e.g., RIP, CLIP, etc.)<br>- qRT-PCR for known targets as readout
**RIP**	*In vivo*, protein as bait	- Without bait protein or with an RNA-binding–impaired mutant of the bait protein<br>- If the bait protein has a tag (e.g., GFP), an IP using tissue expressing only the tag, without the fused protein of interest, in the same cellular compartment is recommended	- Validation of bound RNAs with other *in vitro* methods (e.g., EMSA, ITC, etc.)<br>- Comparison with other *in vivo* methods such as CLIP<br>- qRT-PCR for known targets as readout
**CLIP (iCLIP, eCLIP, UV-CLAP)**	*In vivo*, protein as bait	- No-crosslink (-UV) control<br>- Without bait protein or with an RNA-binding–impaired	- Validation of bound RNAs with other *in vitro* methods (e.g., EMSA, ITC, etc.)<br>- Comparison with other *in vivo* methods such as RIP

		mutant of the bait protein<br>- If the bait protein has a tag (e.g., GFP), an IP using tissue expressing only the tag, without the fused protein of interest, in the same cellular compartment is recommended	- Motif enrichment analysis
**TRIBE / HyperTRIBE**	*In vivo*, protein as bait	- Catalytically inactive (“dead”) ADAR fusion<br>- ADAR without RBP<br>- WT no-enzyme control<br>- Bait protein with impaired RNA-binding ability	- Validation of bound RNAs with other *in vitro* methods (e.g., EMSA, ITC, etc.)<br>- Comparison with other *in vivo* methods such as RIP or CLIP
**STAMP**	*In vivo*, protein as bait	- Catalytically inactive (“dead”) APOBEC1 fusion<br>- APOBEC1 without RBP<br>- WT no-enzyme control<br>- Bait protein with impaired RNA-binding ability	- Validation of bound RNAs with other *in vitro* methods (e.g., EMSA, ITC, etc.)<br>- Comparison with other *in vivo* methods such as RIP or CLIP
**PUP-tagging**	*In vivo*, protein as bait	- Catalytically inactive (“dead”) PUB-2 fusion<br>- PUB-2 without RBP<br>- WT no-enzyme control<br>- Bait protein with impaired RNA-binding ability	- Validation identified RNAs with other *in vitro* methods (e.g., EMSA, ITC, etc.)<br>- Comparison with other *in vivo* methods such as RIP or CLIP
**APEX2 proximity labeling**	*In vivo*, protein as bait	- APEX2 alone (which should be localized in the same compartment as the protein of interest)<br>- APEX2 fused to the protein of interest but with impaired RNA-binding ability<br>- No-biotin control	- Validation of bound RNAs with other *in vitro* methods (e.g., EMSA, ITC, etc.)<br>- Comparison with other *in vivo* methods such as RIP or CLIP

References

Dobin A, Davis CA, Schlesinger F, Drenkow J, Zaleski C, Jha S, Batut P, Chaisson M, Gingeras TR (2013) STAR: ultrafast universal RNA-seq aligner. Bioinformatics 29: 15–21

Hackl T, Hedrich R, Schultz J, Förster F (2014) proovread: Large-scale high-accuracy PacBio correction through iterative short read consensus. Bioinformatics 30: 3004–3011

Koren S, Walenz BP, Berlin K, Miller JR, Bergman NH, Phillippy AM (2017) Canu: scalable and accurate long-read assembly via adaptive k-mer weighting and repeat separation. Genome research 27: 722–736

Li H (2018) Minimap2: pairwise alignment for nucleotide sequences. Bioinformatics 34: 3094–3100

Li X, Li D (2019) An improved collaborative filtering recommendation algorithm and recommendation strategy. Mobile Information Systems 2019: 3560968

Moldován N, Tombácz D, Szűcs A, Csabai Z, Snyder M, Boldogkői Z (2018) Multi-platform sequencing approach reveals a novel transcriptome profile in pseudorabies virus. Frontiers in Microbiology 8: 2708

Raghavachari N, Garcia-Reyero N (2018) Overview of gene expression analysis: Transcriptomics. In: Raghavachari N, Garcia-Reyero N (eds) Gene Expression Analysis: Methods and Protocols. Springer New York, New York, NY, pp. 1–6

Rhoads A, Au KF (2015) PacBio sequencing and its applications. Genomics, Proteomics & Bioinformatics 13: 278–289

de la Rubia I, Srivastava A, Xue W, Indi JA, Carbonell-Sala S, Lagarde J, Albà MM, Eyras E (2022) RATTLE: reference-free reconstruction and quantification of transcriptomes from Nanopore sequencing. Genome Biology 23: 153

Salmela L, Rivals E (2014) LoRDEC: accurate and efficient long read error correction. Bioinformatics 30: 3506–3514

Tseng E, Qin X, Raveendran M, Liu Y, Murali SC, Han, Worley KC, Rogers J, Hon T, Clark T, Tublitz N (2016) Cogent: Reconstructing the coding genome using full-length transcriptome sequences without a reference. Plant & Animal Genome Conference XXIV

Wang B, Regulski M, Tseng E, Olson A, Goodwin S, McCombie WR, Ware D (2018) A comparative transcriptional landscape of maize and sorghum obtained by single-molecule sequencing. Genome Research 28: 921–932

Wu TD, Watanabe CK (2005) GMAP: A genomic mapping and alignment program for mRNA and EST sequences. Bioinformatics 21: 1859–1875

Zhang G, Sun M, Wang J, Lei M, Li C, Zhao D, Huang J, Li W, Li S, Li J, Yang J, Luo Y, Hu S, Zhang B (2019) PacBio full-length cDNA sequencing integrated with RNA-seq reads drastically improves the discovery of splicing transcripts in rice. The Plant Journal 97: 296–305

Zhao L, Zhang H, Kohnen MV, Prasad KVSK, Gu L, Reddy ASN (2019) Analysis of transcriptome and epitranscriptome in plants using PacBio Iso-Seq and nanopore-based direct RNA sequencing. Frontiers in Genetics Volume 10-2019:
